# Endovascular treatment of hepatic arterioportal fistula complicated with giant portal vein aneurysm via percutaneous transhepatic US guided hepatic artery access: a case report and review of the literature

**DOI:** 10.1186/s42155-019-0084-y

**Published:** 2019-11-20

**Authors:** Umut Oguslu, Sadik Ahmet Uyanik, Burçak Gümüş

**Affiliations:** Department of Radiology, Okan University Hospital, Aydinli Cad. No: 2 Okan Universitesi Hastanesi Icmeler, Tuzla, Istanbul Turkey

**Keywords:** Hepatic arterioportal fistula, Percutaneous transhepatic access, Amplatzer vascular plug

## Abstract

**Background:**

Hepatic arterioportal fistulas are rare, abnormal, direct communications between hepatic artery and portal venous system. Treatment options shifted from surgery to endovascular interventions. Catheterization may be challenging. We report a case of a hepatic arterioportal fistula treated successfuly with Amplatzer Vascular Plug II via percutaneous transhepatic hepatic artery access after failed transfemoral approach.

**Case presentation:**

58 year old woman presented with right heart failure, kidney insufficiency and massive ascites related to portal hypertension caused by hepatic arterioportal fistula. She had a history of previous abdominal surgery. Colour Doppler ultrasound and computed tomography revealed a giant portal vein aneurysm related to large hepatic areterioportal fistula. Endovascular treatment was planned. Catheterization of the hepatic artery could not be realized due to severe tortuosity and angulation of the celiac artery and its branches. Access to the hepatic artery was obtained directly via percutaneous transhepatic route and fistula site was embolized with Amplatzer Vascular Plug II and coils. Immediate thrombosis of the aneurysm sac and draining portal vein was observed. Patients clinical status improved dramatically.

**Conclusion:**

Transcatheter embolization is the first choice of the treatment of hepatic arterioportal fistulas but the type of the therapy should be tailored to the patient and interventional radiologist should decide the access site depending on his own experience if the routine endovascular access can not be obtained.

## Background

Arterioportal fistulas (APFs) are rare vascular anomalies that consist of direct connection between mesenteric arterial structures to the portal veins. Majority of the APFs are asymptomatic. They may manifest with gastrointestinal bleeding, ascites, high – output heart failure, diarrhea. Portal hypertension and hepatic cirrhosis (Vauthey et al. 1997). The interval between fistula formation and its detection has a range between hours to decades (Ryan and Lorber 1968).

Open partial hepatic resection or surgical ligation of the feeding artery for treatment is the surgical option but has high risk of morbidity and long hospital stay in this patient with severe comorbidities. Recently endovascular treatment has become the first choice of treatment because of its less invasive nature and higher success rate in selected cases (Hirakawa et al. 2013).

Target vessel anatomy may preclude safe access to APF localization via transfemoral approach. In these situations interventionalists should be aware of different access sites. We represent a case of hepatic APF treated successfully via percutaneous transhepatic hepatic artery access after failed antegrad catheterization.

## Case report

A 58 year old female patient was admitted to a local hospital complaining of abdominal swelling, right upper quadrant pain, bruit, dyspnea, effort intolerance and diffuse oedema formation. The patient had no history of alcohol abuse but a history of ovarian cyst surgery and multiple pregnancies.

Physical examination revealed a large volume of ascites. Echocardiography showed cardiomegaly and signs of high – output heart failure. Ultrasound (US) and Computed Tomography (CT) scan demonstrated a large APF between replaced left hepatic artery of the left gastric artery draining into the left portal vein causing a giant aneurysm formation.

Phsyical and labarotory examination was as follows; pulse rate was 80 beats/min and arterial blood pressure was 160/90 mmHg, no sign of jaundice. Liver function tests were within normal limits. Serum creatitine level was 2,5 mg/dl (normal range 0.5–1.1 mg/dl). Hepatitis viral markers were negative for HBV, HCV and HIV.

**Fig. 1:**
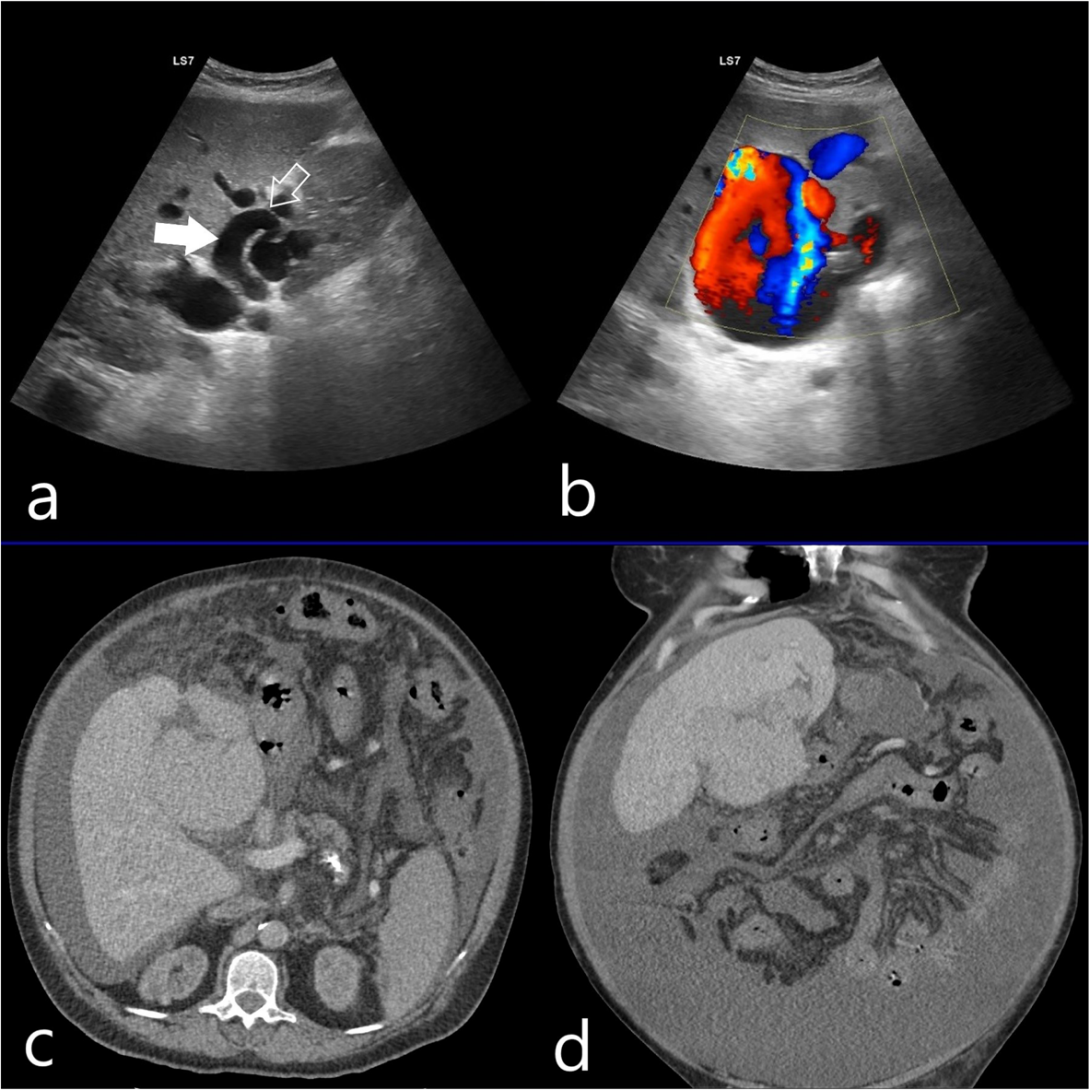
RDUS view (**a** and **b**) showing the direct connection (hollow white arrow) between dilated hepatic artery (solid white arrow) and portal vein (**a**); Large saccular aneurysm formation between left hepatic artery and portal vein (**b**). Portal phase CT (**c**,**d**) confirms US findings and demonstrates massive ascites

US examination demonstrated massive ascites, macrolobulation of the liver contour demonstrating chronic liver disease, splenomegaly (16 cm) and large tubuler structures in the left lobe of the liver. Color Doppler Ultrasound (CDUS) showed direct arteriovenous fistulae between dilated left hepatic artery and left portal vein. There was a giant saccular aneurysm (130x90mm) originating from left portal vein. Right portal vein was dilated too and main portal vein showed hepatopedal flow direction (Fig. 1a,b). Triphasic computed tomography comfirmed US and RDUS findings (Fig. 1c,d).

**Fig. 2:**
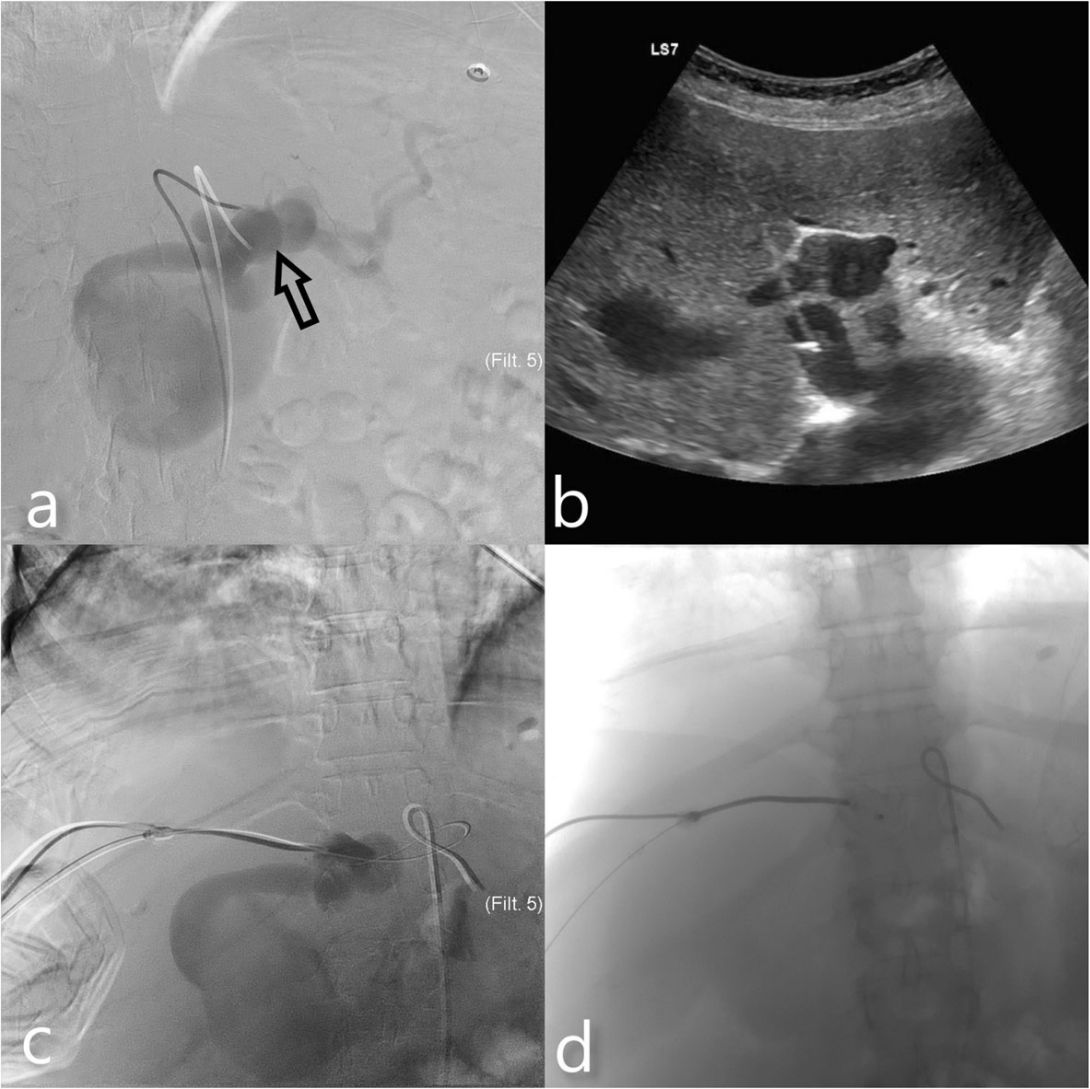
Celiac artery angiogram (**a**) showing the APF location (hollow black arrow). The aneurysm also fills at early phase of the contrast media injection US image (**b**) showing the needle localization during percutaneous transhepatic puncture of left hepatic artery. **c** Contrast injection through hepatic access shows exact localization of the vascular sheath (**d**) Amplatzer vascular plug II deployment

A diagnostic celiac angiography was initially performed. Celiac artery was selectively catheterized with 5F Simmons 1 catheter (Terumo, Leuven, Belgium). Angiography showed the direct fistulae between the left hepatic artery and the left portal vein through single large window (Fig. 2a). The selective catheterization of the left hepatic artery could not be realized despite all attempts because of the severe tortuosity and angulation of the celiac and common hepatic artery. 8F catheter was placed in the right lower quadrant of the peritoneal cavity and a total of 24,000 cc (6000 cc/d) ascites fluid was drained prior to the next session.

**Fig. 3:**
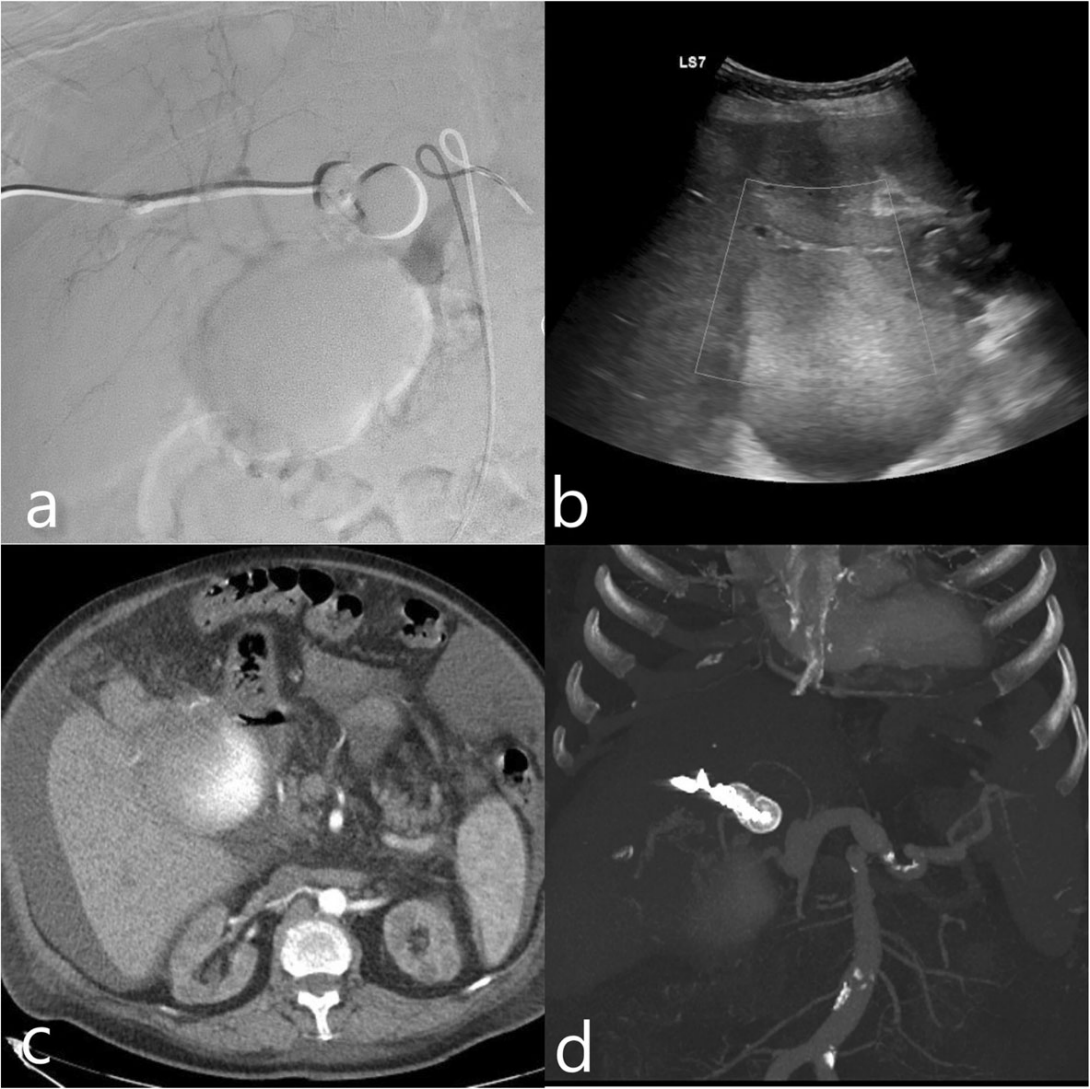
Celiac angiogram and US images (**a**-**b**) showing immediate thrombosis formation in the portal vein and aneurysm sac while preserving hepatic artery circulation. One month follow up triphasic CT(**c**-**d**); arterial phase shows no filling of the portal vein and aneurysm sac following the AVP II; coils deployed through the access site can be seen

At the following session an US guided percutaneous transhepatic puncture of the left hepatic artery was planned. We eliminate the option of transhepatic portal venous access because of the aneurysmal dilation of the portal vein, the risk of AVP migration was relatively high. At first step, a celiac artery catheterization was performed and baseline angiograms were obtained. Doppler examination demonstrated that portal vein showed aneurysmal dilation (33 mm) and tortuous course immediately after the fistula localisation. Hepatic artery showed straight course and smaller diameter (17 mm). Arterial access was achived under US guidance with AccuStick II introducer system (Boston Scientific, Marlborough, USA). After arterial puncture with 21G needle 0.018″ guidewire was advanced (Fig. 2b). System was upsized to exhange 0.035″ guidewire and 7F introducer sheath (Boston Scientific, Marlborough, USA) was advanced into the hepatic artery. After check angiograms were obtained (Fig. 2c), 22x18mm Amplatzer II vascular plug (AVP II) (AGA Medical Corporation, Plymouth, MN, USA) was deployed through the vascular sheath in the left hepatic artery proximal to the fistula localization (Fig. 2d). Coil embolization was performed for the complete occlusion of the remnant sac and the access tract with 10x14mm and 10x30mm Complex Helical pushable coils (Boston Scientific, Marlborough, USA). Completion angiography images demonstrated complete cessation of flow through the fistula (Fig. 3a). US showed immediate thrombosis in the draining portal vein segment and the aneurysm sac distal to the fistula location (Fig. 3b).

Patient’s status was stable during the postoperative period. Liver function tests showed no abnormality, and serum creatinin level decreased dramatically. First month follow up laboratory tests were normal and check CT angiography showed complete cessation of flow and no filling of the aneurysm was observed (Fig. 3c,d). The patients is now at 9. month follow up, she is symptom free, her blood test are within normal range. Check RDUS and CTA showed totally thrombosed aneurysmal sac and portal flow was preserved.

## Discussion

When APF is clinically suspected CDUS is the first step of diagnosis. A triphasic CT scan can detect more centrally located lesion. Finally angiography is the gold standart diagnostic tool but should be kept in mind when intervention is planned (Vauthey et al. 1997; Bolognesi et al. 2000).

Guzman et al. proposed a classification system for APFs (Guzman et al. 2006). Our case was more likely to be classified as Type 2. Patient had no history of blunt or penetrating trauma but she had gynecologic operation and multiple pregnancies. APF of this patient led to parenchymal liver disease and resulted with portal hypertension symptoms (Zuidema et al. 1963). Hyperdynamic blood flow was complicated with congestive heart failure and prerenal kidney failure.

Architecture of the fistula determines the choice of the embolic agent. It depends on the size and number of the feeding artery, accessibility of the fistula location. Gelfoam, steel coils, detachable balloons and combination of microcoils with N-butyl 2-cyanoacrylate are effective choices of treatment. Coils are the most commonly used embolic agents (Tasar et al. 2005; B. E. Cil 2004; Botelberge et al. 2005).

If the fistula is greater than 8 mm and high flow rate, arterial embolization is controversial due to risk of nontraget embolization, arterial thrombosis and coil migration is now the possible complications. The AVP II (St. Jude Medical, Plymouth, Minn) has been widely used in many clinical situations (Wang et al. 2012; B. Cil et al. 2006; Jang et al. 2013; Roux et al. 2009). It can occlude a large vessel with a single device in one step. This device has some disadvantages too. It is required at least 4F sheath or 5F guiding catheter or sheath for delivery. The release wire is relatively stiff so it may be difficult to advance and release. So target vessel should have a straight segment and constant diameter (Tzilalis et al. 2011; Wang et al. 2012). In our case we used 22 mm diameter AVP II plug so we needed 7F sheath to deploy the device.

The vascular access to reach the treatment zone can be a challenging issue in the endovascular management of mesenteric aneurysms and fistulae. The target vessel may have tortuosity and elongation due to hemodynamic changes created by the hyperdynamic flow. As far as our knowledge, this is the first case reported in the English literature performed by direct puncture of the hepatic artery. When an access problem to reach the target vessel arises, percutaneous route should be considered as an alternative route for the treatment. It should not be a routine access but when an endovascular access can not be obtained, a need for a different approach comes into consideration. Perihepatic ascites should be drained first that will shorten the distance between target vessel and abdominal wall to assure a more stable access. In our case a total of 24,000 cc ascites was drained prior to the second session. Although it has many potential risks (peritoneal hemorrhage, arterio-biliary/biliary-venous fistula, pneumothorax), direct puncture of the diseased segment can be safely performed under sonographic guidance. Direct visualization of the needle path will significantly decrease complication rates. AVP is a valuable device to get sufficient occlusssion in these dilated segments. Percutaneous transhepatic route provides a short and straight access that will facilitate advancing large diameter AVPs through the sheath easily.

## Conclusion

To our knowledge this is the first case in English literature that a hepatic APF is treated by endovascular approach through a percutaneous transhepatic arterial access. In our case transfemoral approach to the hepatic artery could’t be realized because of the severe tortuosity and angulation of the celiac artery that is the main technical failure in the endovascular management of hepatic and mesenteric aneurysms and fistulae. Transcatheter embolization is the first choice of the treatment of APF but the type of the therapy should be tailored to the patient and interventional radiologist should decide the access site depending on his own experience if the routine endovascular access can not be obtained.

## Data Availability

Data sharing not applicable to this article as no datasets were generated or analysed during the current study.

## Abbreviations

APF: Arterioportal fistula
AVP: Amplatzer vascular plug
CDUS: Color Doppler Ultrasound
CT: Computed Tomography
US: Ultrasound
